# Characteristics of pediatric thoracic trauma: in view of before and after the establishment of a regional trauma center

**DOI:** 10.1007/s00068-021-01658-4

**Published:** 2021-04-03

**Authors:** Pil Young Jung, Jae Sik Chung, Youngin Youn, Chang Wan Kim, Il Hwan Park, Oh Hyun Kim, Chun Sung Byun

**Affiliations:** 1 Regional Trauma Center, Wonju Severance Christian Hospital, Wonju, Republic of Korea; 2 Department of Surgery, Yonsei University Wonju College of Medicine, Wonju, Republic of Korea; 3 Department of Thoracic and Cardiovascular Surgery, Yonsei University Wonju College of Medicine, 20 Ilsan-ro, Wonju, Gangwon 26426 Republic of Korea; 4 Department of Emergency Medicine, Yonsei University Wonju College of Medicine, Wonju, Republic of Korea

**Keywords:** Pediatric, Thoracic trauma, Thoracic injury, Trauma center, Injury severity score

## Abstract

**Purpose:**

Pediatric thoracic trauma differs from those of adult in terms of the small anatomy and rapid tissue recovery. Therefore, it is important to know the characteristics of the pediatric thoracic trauma to improve treatment results. In addition, this study examined the changes in pediatric thoracic trauma features and results from the establishment of a level 1 regional trauma center.

**Methods:**

Data of 168 patients’ ≤ 15 years old diagnosed with thoracic trauma between 2008 and 2019 were retrospectively analyzed.

**Results:**

Pedestrian traffic accidents were the most common cause of chest injury. The average injury severity score was 17.1 ± 12.4 and the average pediatric trauma score was 5.6 ± 4.1. Lung contusion was the most common in 134 cases. There were 48 cases of closed thoracostomy. There was one thoracotomy for cardiac laceration, one case for extracorporeal membranous oxygenation, and six cases for embolization. Of all, 25 patients died, providing a mortality rate of 14.9%. In addition, independent risk factors of in-hospital mortality were hemopneumothorax and cardiac contusion. Since 2014, when the level 1 regional trauma center was established, more severely injured thoracic trauma patients came. However, the mortality was similar in the two periods.

**Conclusions:**

Understanding the clinical features of pediatric thoracic trauma patients can help in efficient treatment. In addition, as the severity of pediatric thoracic trauma patients has increased due to the establishment of the regional trauma center, so pediatric trauma center should be organized in regional trauma center to improve the outcomes of pediatric thoracic trauma.

**Supplementary Information:**

The online version contains supplementary material available at 10.1007/s00068-021-01658-4.

## Background

Trauma in children under the age of 15 is the most common cause of disability and mortality in the pediatric population, excluding diseases [1]. Among them, thoracic trauma in children is uncommon, but it can result in minor to very fatal injuries [2]. The anatomical structure of children is characterized by a large head, a small body, soft bones, undeveloped muscles, and rapidly recovering tissues [1, 3]. These have different effects on head, chest, abdomen, pelvic and bone injuries in pediatric trauma. According to statistics from the Republic of Korea, accidents are the second leading cause of death between the ages of 1 and 15 [4]. In the case of such pediatric trauma, it is desirable to organize the pediatric trauma center to treat pediatric trauma properly, but it is impossible to establish and operate the separate pediatric trauma center by investing enormous economic and manpower resources in Korea. Most surgical resident in Korea trained the pediatric treatment during their training period for congenital disease management of pediatric patients. However, there is no appropriate training system of pediatric trauma. In addition, most physician does not want to train of trauma, because of low quality of life and relatively low income. For this reason, pediatric traumatologists are of no interest. In addition, there is also a problem in treating pediatric trauma at the emergency center that specializes in treating pediatric diseases, but not prepared for severe pediatric injuries As an alternative to this, the level 1 regional trauma center can be considered to provide medical treatment for pediatric trauma as well as adults. Since its establishment supported by the Korean Ministry of Health and Welfare (KMHW), it conducts inspections of each level 1 regional trauma center’s resources, and treatment quality once every 3–4 months. For this reason, it is thought that there will be differences in the treatment of pediatric thoracic trauma after the trauma center was established.

The first objective of this study was to determine the distribution of the type of trauma, treatment, and outcome of patients with pediatric thoracic trauma patients under the age of 15 who visited the emergency center. In addition, since 2014, our hospital has established the level 1 regional trauma center in the emergency center, with the securing of a trauma team to provide prompt treatment. Therefore, the second objective of this study is to analyze changes in pediatric thoracic trauma patients before and after the establishment of the regional trauma centers.

## Materials and methods

This study included children under 15 years of age admitted to the emergency center in Wonju Severance Christian Hospital for chest injuries, from January 2008 to December 2019. Children who came to the hospital with simple chest wall contusion were excluded from this study. Children were divided into age groups 0–3 year, 4–7 year, 8–11 year, and 12–15 year for comparison. Chest injury was diagnosed by X-ray and computed tomography (CT). Bone scan was not performed specifically to diagnose rib fractures. Chest CT was actively performed in cases of suspected comorbid damage in the mediastinum or thoracic cavity, but not in cases of simple fracture or low severity injury. Hematological tests were performed to obtain a baseline, with complete blood counts, blood chemistry, a prothrombin/a partial thromboplastin time, etc. and also creatine kinase MB isoenzyme (CK-MB)/Troponin I for cardiac enzymes. An electrocardiogram (EKG) was performed to confirm cardiac injury. There are no exact diagnostic criteria for cardiac contusion in the EKG, but according to the cardiac injury scale of the American Association for the Surgery of Trauma (AAST), non-specific ST changes, premature atrial or ventricular contractions, persistent sinus tachycardia, heart block, and ischemic changes of EKG were defined evidence of the cardiac injury [5]. Those change of EKG with significant Troponin I elevation were accepted for cardiac contusion in this study.

Indication of mechanical ventilation was respiratory failure, cardiac insufficiency, and neurologic dysfunction with GCS < 8 due to trauma. If the chest injury was severe, or required mechanical ventilation assistance, these patients were treated in the department of the thoracic and cardiovascular surgery. If the chest injury was not severe, or if surgery was necessary due to other associated injuries, patients were admitted to other department depending on the associated injury. The imaging tests of all patients were analyzed, and multidisciplinary discussions were conducted to record their injury severity score (ISS) and pediatric trauma score (PTS). We analyzed the overall causes and manifestations of chest injury in children, treatment methods and follow-up, and evaluated the independent risk factors for death due to thoracic trauma in children. In addition, the differences in patient characteristics and prognosis before and after the establishment of the regional trauma center were compared.

IBM SPSS version 25.0 (SPSS Inc., Chicago, USA) was used for statistical analysis. The *χ*^2^ test and Fisher’s exact test were used to compare medians for categorical variables. The Student’s *t* test and Mann–Whitney test was used to compare means for continuous variables. Logistic regression analysis was used to identify independent factors associated with in-hospital mortality. The significance level was set at a *p* value < 0.05. Quantitative data are shown as mean ± standard deviation or with median and range, unless indicated otherwise.

## Results

### Characteristics of pediatric thoracic trauma patients

**Fig. 1:**
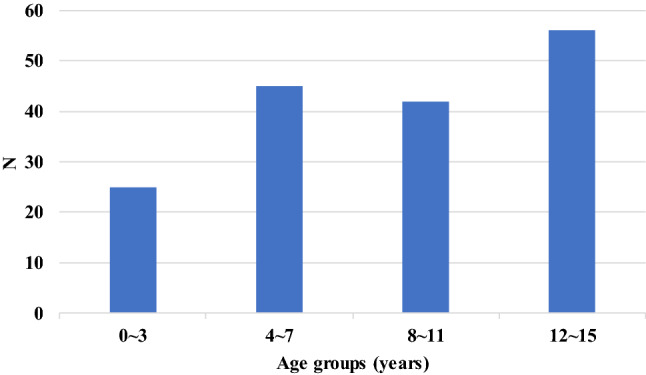
Thoracic trauma cases by age groups. *N* number

**Fig. 2:**
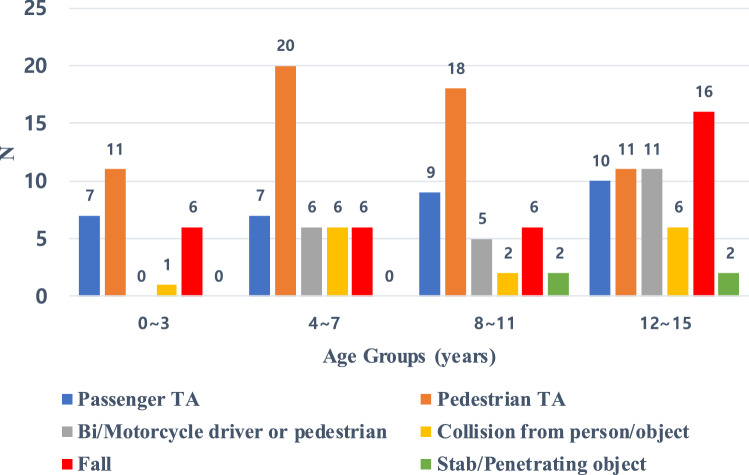
Cause of thoracic trauma by age groups. *N* number, *TA* traffic accident

There was a total of 168 pediatric thoracic trauma patients during the study period. Among them, 108 were male, and the mean age was 8.7 ± 4.4 years (0–15 years old, median 9 years). There were 56 children in 12–15 age group, more than other age groups (Fig. 1). Pedestrian traffic accidents were the most common cause of chest injury, seen in 60 cases, followed by falls in 34 cases, automobile passenger accidents in 33 cases, bicycle/motorcycle accidents in 22 cases, a collision from a person or object in 15 cases, and a stabbing or piercing injury in 4 cases (Table 1). The average ISS was 17.1 ± 12.4, and an ISS score of 9 or higher present in 125 patients (74.4%). The average PTS was 5.6 ± 4.1, and 121 patients had 8 points or less, accounting for 72.0%. Overall, pedestrian traffic accidents were more common than other accidents, but according to age groups, falls were most common in the 12–15 years old group (Fig. 2).

**Table 1:** Demographic characteristics of pediatric thoracic trauma patients

Variables	*N* (%)
Sex	
Male	108 (64.3)
Age	
0–3	25 (14.9)
4–7	45 (26.8)
8–11	42 (25.0)
12–15	56 (33.3)
Mechanism of injury	
Pedestrian TA	60 (35.7)
Fall	34 (20.2)
Passenger TA	33 (19.6)
Bi/motorcycle driver or passenger	22 (13.1)
Collision from person/object	15 (8.9)
Stab/penetrating object	4 (2.4)
ISS	
1–8	43 (25.6)
9–15	42 (25.0)
16–25	50 (29.8)
26–40	27 (16.1)
41–75	6 (3.6)
PTS	
9–12	47 (28.0)
5–8	62 (36.9)
1–4	38 (22.6)
−6–0	21 (12.5)

*N* number, *TA* traffic accident, *PTS* pediatric trauma score, *ISS* injury severity score

Clinically, lung contusion was the most common finding with 134 cases, pneumomediastinum with 87 cases, pneumothorax with 75 cases, rib fracture with 52 cases, hemothorax with 40 cases, hemopneumothorax with 29 cases, traumatic subcutaneous emphysema with 22 cases, sternal fractures with 7 cases, cardiac contusion with 8 cases, cardiac laceration with one case, and right subclavian artery injury with one case (Table 2). Associated injuries included cerebral injury in 91 cases, abdominal injury (liver, spleen laceration, etc.) in 83 cases, extremity injuries in 70, facial injuries in 47, spinal injuries in 22, and pelvic injuries in 21 cases (Table 3).

**Table 2:** Spectrum of thoracic injuries in pediatric thoracic trauma patients

Region	Injury type	*N* (%)
Lung	Contusion	134 (79.8)
	Pseudocyst	11 (6.5)
	Asphyxia	2 (1.2)
Pleura	Pneumothorax	75 (44.6)
	Hemothorax	40 (23.8)
	Hemopneumothorax	29 (17.3)
Mediastinum	Pneumomediastinum	8 (4.8)
	Hemomediastinum	4 (2.4)
	Tracheal laceration	1 (0.6)
	Diaphragm rupture	1 (0.6)
Chest wall	Rib fracture	52 (31.0)
	Sternum fracture	7 (4.2)
	Flail chest	3 (1.8)
	Subcutaneous emphysema	22 (13.1)
	Chest wall laceration	9 (5.4)
Heart	Cardiac contusion	8 (4.8)
	Cardiac laceration	1 (0.6)
Vessel	Subclavian artery tear	1 (0.6)

*N* number

**Table 3:** Inured body regions other than thorax

Region	*N* (%)
Cerebral injury	91 (54.2)
Abdominal injury	83 (49.4)
Extremity injury	70 (41.7)
Facial injury	47 (28.0)
Spinal injury	22 (13.1)
Pelvic injury	21 (12.5)

*N* number

### Treatment results of pediatric thoracic trauma patients

In Table 4, there were 48 cases of closed thoracostomy due to chest injury in the emergency room (ER). There were 43 cases which required mechanical ventilation. Among them, 15 (8.9%) were treated with mechanical ventilation for severe chest injuries such as circulatory arrest due to cardiac or respiratory cause, airway protection from massive hemoptysis, and inadequate oxygenation due to severe lung contusion. The others treated mechanical ventilation started when GCS < 8 due to hemorrhagic shock, or severe brain injury. Fifteen patients who received mechanical ventilation support due to chest injury, 11 died. Of the 11 people who died, 7 died from severe chest injury, two died from intractable bleeding, and two people died due to the accompanying brain injury, although their lung damage improved. The mean duration of mechanical ventilator assistance of four surviving patients was 3.5 ± 1.0 days. Their lung condition was quickly improved, and oxygenation was well maintained, and the ventilator could be ended. Sixteen patients were admitted to the ER and underwent cardiopulmonary resuscitation (CPR): however, ten died in the ER, five died after admission to the intensive care unit (ICU), and one patient was successfully discharged after treatment. Emergency surgery or procedure was performed in 22 cases as follows: exploratory laparotomy in 7 cases, cranial decompression in 7 cases, vascular embolization on the facial, liver, or spleen bleeding in 6 cases, veno-venous extracorporeal membranous oxygenation (ECMO) in one case and exploratory thoracotomy in one case. Exploratory thoracotomy was performed in a patient with multiple stab wounds, and he received suture repair for his left ventricular myocardium laceration and lung laceration.

**Table 4:** Procedures according to injury site in pediatric thoracic trauma patients

Variables	*N* (%)
Emergency room procedure	
Closed thoracostomy	48 (28.6)
Mechanical ventilation	43 (25.6)
Mechanical ventilation by chest injury	15 (8.9)
Cardio-pulmonary resuscitation	16 (9.5)
Emergency procedure	
Exploratory laparotomy	7 (4.2)
Cranial decompression	7 (4.2)
Embolization	6 (3.6)
ECMO	1 (0.6)
Exploratory thoracotomy	1 (0.6)
Elective procedure	
Extremity surgery	22 (13.1)
Facial surgery	11 (6.5)
Spinal surgery	6 (3.6)

*N* number, *ECMO* extracorporeal membranous oxygenation

In Table 5, the average hospitalization period was 16.1 ± 25.1 days, and the ICU stay was 8.5 ± 19.1 days. Ninety-eight were discharged home, and 30 were discharged to the rehabilitation facility. Fifteen patients were transferred to other hospitals during hospitalization for various reasons. Of the total trauma patients, 25 died, giving a mortality rate of 14.9%, but among them, 8 patients died due to thoracic trauma. By age groups, nine people died in the 12–15 years old group, which showed the highest mortality rate. In a view of trauma score, 38 patients had an ISS score of 25 or higher, 21 of whom died, resulting in a 55.3% mortality rate; and 21 patients with a PTS score of 0 or lower, 16 of whom died, resulting in 76.2% mortality rate.

**Table 5:** Outcomes of pediatric thoracic trauma patients

Variables	*N* (%)
Interval day	
Hospital stay	16.1 ± 25.1
ICU stay	8.5 ± 19.1
Discharge type	
Home	98 (58.3)
Rehabilitation facility	30 (17.9)
Transfer to other hospital	15 (8.9)
Expired	25 (14.9)
Main cause of death	
Craniofacial injury	10 (6.0)
Thoracic injury	8 (4.8)
Abdominal injury	7 (4.2)
Death incidence according to age	
0–3	5
4–7	4
8–11	7
12–15	9

Data are presented as *n* (%) or mean ± standard deviation

*N* number, *ICU* intensive care unit

### Risk factors for predicting death in pediatric thoracic trauma patients

Univariable analysis revealed that the statistically significant following factors were associated with in-hospital mortality: hemothorax; hemopneumothorax; subcutaneous emphysema; cardiac contusion (*p* < 0.05). Multivariable analysis identified that hemopneumothorax and cardiac contusion were the independent risk factors for in-hospital mortality with high odds ratio (Table 6).

**Table 6:** Multivariable logistic regression for predictor of mortality in pediatric thoracic trauma

Variables	Odds ratio (95% CI)	*p* value
Hemothorax	1.162 (0.248–5.441)	0.848
Hemopneumothorax	8.968 (3.295–24.411)	< 0.001
Subcutaneous emphysema	1.224 (0.340–4.403)	0.757
Cardiac contusion	19.044 (3.577–101.391)	0.001

*CI* confidence interval

### Changes in pediatric thoracic trauma patients before and after the establishment of the regional trauma center

From 2008 to 2019, 487,981 patients were admitted to the emergency center. Of these admissions, 33,645 trauma cases were reported, and 1358 pediatric trauma patients under the age of 15 years were identified. Among them, only 168 children were diagnosed with pediatric thoracic trauma, and the frequency was 12.4% in pediatric trauma and 0.5% in all trauma patients. In this study, characteristics of pediatric thoracic trauma can also be identified, but there was a significant change with the establishment of a level 1 regional trauma center in 2014, during the study period. As shown in Table 7, the ISS and abbreviated injury scale (AIS) of chest showed more severe injured patients after the regional trauma center was established. In the injury variables, the incidence of facial injuries and serum troponin I levels increased, but the incidence of pneumothorax decreased. Since the establishment of the regional trauma center, the number of patients admitted to the ICU and the number of patients transferred to the rehabilitation facility upon discharge have increased. In the cases of PTS and mortality, it was not statistically significant.

**Table 7:** Differences of the pediatric thoracic trauma patient before and after establishment of a trauma center

Variables	Before trauma center (*n* = 85)	After trauma center (*n* = 83)	*p* value
Trauma score			
ISS	**14.8 ± 10.4**	**19.2 ± 13.8**	**0.018**
AIS of chest	**2.6 ± 0.9**	**2.91 ± 1.0**	**0.036**
PTS	6.14 ± 4.2	5.04 ± 4.0	0.066
Associated injury			
Cerebral injury	42	49	0.220
Facial injury	**17**	**30**	**0.025**
Abdominal injury	46	37	0.222
Pelvic injury	13	8	0.352
Spine injury	11	11	1.000
Extremity injury	35	35	1.000
Thoracic trauma			
Pneumothorax	**45**	**30**	**0.031**
Hemothorax	16	24	0.206
Hemopneumothorax	13	16	0.684
Rib fracture	30	22	0.245
Multiple rib fracture	20	10	0.069
Sternum fracture	2	5	0.443
Lung contusion	64	70	0.446
Cardiac contusion	2	6	0.165
Troponin I elevation	**18**	**30**	**0.040**
Procedure			
CPR	5	11	0.188
Embolization	2	4	0.440
Closed thoracostomy	24	24	1.000
Admission to			
OR	5	9	0.276
ICU	**39**	**58**	**0.002**
Ward	38	35	0.758
Discharge to			
Home	49	49	1.000
Rehab facility	**10**	**20**	**0.045**
Mortality			
Overall	10	15	0.387

*ISS* injury severity score, *AIS* abbreviated injury scale, *PTS* pediatric trauma score, *ER* emergency room, *CPR* cardiopulmonary resuscitation, *ICU* intensive care unit

Bold value indicates statistically significant difference, with *p* < 0.05

## Discussion

Wonju is the largest city in Gangwon-do, the Republic of Korea, with a population of approximately 350,000. In addition, due to the characteristics of Gangwon-do, Wonju is the surrounding region consists of mountainous areas. The distance traveled between the surrounding cities is lengthy, and in summer and winter, it is a city with a large floating population for recreation and sports. Wonju Severance Christian Hospital, which is the tertiary referral hospital in Wonju, is currently designated as a regional emergency center, and is always responding to the outbreak of emergency patients that occur in a population of about 2.5 million people in a treatment area covering nearly 100 km. The emergency center is visited by 40,000 patients every year, of which almost 2800 are trauma patients. Most of them are traffic accident, falls, and sports injuries victims, of which nearly 110 are pediatric trauma patients under the age of 15 in every year. In this study, the average number of pediatric patients with thoracic trauma was 14 per year.

### Known anatomical and behavioral characteristic of pediatric thoracic trauma

Our study showed similar results to previous research, the incidence of thoracic trauma is higher mainly in boys because of increasing activity by age [6]. The number of pedestrian traffic accidents between the ages of 0 and 11 was high, but the number of falls was highest between the ages of 12 and 15. This demonstrates that younger children are more likely to be in a pedestrian traffic accident due to lack of attention, and children over the age of 12, although tending to be more careful, are typically more active and mischievous, displaying a higher fall rate.

Thoracic trauma in children is different than in adults. This is due to the different anatomical features of the pediatric thorax. It is known that pediatric ribs are more flexible and can bend to a large extent [7]. Therefore, it has been reported that the incidence of rib fractures during a blunt chest injury is low, and a more pulmonary contusions appear instead. This pattern is known to persist from infancy to puberty [1]. In this study, pulmonary contusion was found to be as high as 79.8% on average, while rib fractures were observed in less than 31%. In addition, these anatomical features can easily damage the organs in the thoracic cavity clinically, and the frequency of flail chests is low [8]. The low incidence of rib fractures suggests that, on the other hand, in the case of children with rib fractures, there is a high possibility of severe damage to intrathoracic or other organs. According to Garcia et al. in 1990, no one died when there was one rib fracture, but the mortality rate was high, at 42%, when there were two or more [9]. Although not shown in the table, there was no significant difference in mortality in children with multiple rib fractures, but the severity of the patient’s injury was statistically higher (ISS was higher, and PTS was lower, *p* < 0.05). The most common associated injury was a head injury (54.2%), followed by abdominal injury and extremity injury. One of the characteristics of children is that they tend to have a higher head to body ratio, which can predict that craniofacial injury is the most associated injury in severe thoracic trauma patients. In addition, the size of the abdomen was relatively large in the pediatric torso, many associated abdominal injuries occurred.

### Conservative treatment vs. aggressive treatment

In this study, 46 children (27.4%) had pneumothorax. Among them, closed thoracostomy was performed on 23, only 50%. The reason for the less closed thoracostomy is that there were many occult pneumothorax observed on CT, not visible on X-ray [10]. They did not perform closed thoracotomy and were followed up. Hemothorax alone was diagnosed in 11 patients (6.5%), but only 4 cases had a chest tube inserted. It seems that the incidence of rib fractures was not as severe as in adults and the amount of hemothorax was small, so fewer chest tubes were inserted. However, 29 children were diagnosed with hemopneumothorax, and 21 of them managed with closed thoracostomy in ER (72.4%). The reason is that patients diagnosed with hemopneumothorax suffered relatively high-energy chest injury, so closed thoracostomy was often performed in the ER.

Cardiac injury does not yet have a gold-standard diagnostic test [11]. However, according to the Eastern Association of Trauma (EAST) blunt cardiac injury practice guidelines, and AAST blunt cardiac injury guideline, abnormal EKG, cardiac enzyme elevation, newly developed echocardiographic finding, and ischemic lesion on nuclear medicine are key finding of cardiac injury [5, 12]. In this study, blunt cardiac contusion was diagnosed as an EKG abnormality and troponin I elevation, and there was no blunt cardiac injury requiring surgery. Among the AAST cardiac injury scales, one patient showed non-specific ST abnormality, which are changes in EKG of a grade I blunt cardiac injury. Seven children had grade II blunt cardiac injury EKG findings, including anterior/inferior wall ischemia, interventricular block, 1st degree AV block, and lateral infarction [5]. Among troponin I was elevated in 48 patients, with only 8 showing EKG abnormalities. Therefore, eight patients who met both criteria were diagnosed with cardiac contusion. One patient underwent explorative thoracotomy with multiple stab wounds in the chest and abdomen, and he was diagnosed with a myocardial laceration. In all, nine patients (5.4%) suffered cardiac injury, including blunt and penetrating cardiac injury.

When admitted to the ER, a total of 43 patients underwent mechanical ventilation. Of these, 15 (8.9%) were treated with mechanical ventilation for severe chest injuries. The others treated with mechanical ventilation because of GCS < 8 due to hemorrhagic shock, or severe brain injury. As shown in other papers, mechanical ventilation is performed often through other major injury and also the rate of mechanical ventilation for severe chest injury is lower than in adults [13, 14]. In 28 patients who underwent mechanical ventilator due to severe brain injury or hypovolemic shock, 11(39.3%) died from irreversible brain injury or multi-organ failure. However, 11(73.3%) out of 15 patients who underwent mechanical ventilator due to chest injury died. Therefore, the mortality rate was relatively high when ventilator treatment was performed for chest injury.

In our study, embolization was performed in six cases, one for facial bleeding, and five for liver or spleen bleeding. All procedures were performed successfully, but two patients died due to concomitant brain or chest injury during treatment. One patient who underwent VV ECMO was a 10 year old boy who had hypo-oxygenation with severe lung contusion after a pedestrian traffic accident. This patient also died of multi-organ failure after 48 h. Such embolization or ECMO has been widely performed in children whose blood vessel size is secured. Recently, it should be noted that resuscitation is actively being performed with emergency percutaneous techniques such as embolization, ECMO, and resuscitative endovascular balloon occlusion of aorta (REBOA) [15, 16].

### Relatively high mortality to overcome

Death occurred in 25 patients (14.9%), but only 8 (4.8%) died from causes associated with chest injury. This can be confirmed in other papers. Patients who died from chest injuries show a mortality rate of 5%, but it is reported that it rises to 40% when abdominal or head injuries are included [17, 18]. In this study, the mortality rate gradually increased with age, which can be explained by the fact that as children grow, their activity level increases, and their exposure to risk factors also increases. The independent factors associated with mortality in pediatric thoracic trauma patients are hemopneumothorax, and cardiac contusion. It should be noted that these injuries showed a relatively high odds ratio, and all of them suffered from high-energy thoracic damage. Therefore, these were contributed to be independent factors that can affect death, and these patients require more aggressive treatment and surveillance during hospitalization.

### The challenging role of the regional trauma center in pediatric thoracic trauma

This study compared the treatments and outcomes of pediatric thoracic trauma from the time the regional trauma center was established. The trauma centers in Korea have established late, and operated differently from the United States or Europe. In addition, the pediatric trauma center was not even considered in trauma center either [19]. The recent data reported preventable mortality rate in trauma patients of the Korea (from 2012) was 35.2%, which remained high, compared with other Organization for Economic Co-Operation and Development countries [20]. As a national project supported by the KMHW, the government initiated a project to establish the level 1 regional trauma centers nationwide, to reduce preventable trauma mortality less than 20% by 2020, and our hospital has been designated as a regional trauma center since 2012. Helicopter transportation of emergency patients has also begun at the same time. Since 2014, the level 1 regional trauma center was constructed, and a trauma team was formed to begin. After the regional trauma center was completed, most of the severely traumatized patients within a radius of 100 km went to the regional trauma center. From the time of transfer, active resuscitation was performed by emergency medicine physician and emergency medical technician via wireless. In addition, as soon as they arrived at the trauma center, emergency medicine physicians started their initial management according to the advanced trauma life support or pediatric trauma life support guidelines. Simultaneously, it is the responsibility of the emergency medicine physicians trigger trauma team activation according to the trauma team activation criteria. Then, the trauma surgeon, emergency physician, thoracic and cardiovascular surgeon, orthopedic surgeon, and neurosurgeon and other specialists performed treatment together.

In this study, 85 pediatric patients with thoracic trauma before the regional trauma center establishment, and 83 pediatric patients after center establishment, attended at the hospital. After the regional trauma center was established, the ISS and AIS of chest were higher and more severe. In addition, although not statistically significant, PTS has shown that more children with poor scores presented after the regional trauma centers were established. From the Table 7, it can be speculated that more severe trauma patients were admitted after the level 1 regional trauma center was initiated. As the severity of the children’s chest injury increased, associated facial damage increased, according to the characteristics of the children’s anatomy. In addition, troponin I was increased due to severe thoracic damage. In addition, as the trauma ICU was secured more than before the trauma center established, the ICU admission for pediatric thoracic trauma increased. In addition, cases for rehabilitation treatment at discharge upsurged due to severe injured children increased. All of this reason seems to have appeared as the number of severely injured patients increased after the trauma center was established. However, in the results, there is no statistical difference in the mortality rate although increased trauma death after established regional trauma center (before vs. after; 11.8% vs. 18.1%, *p* = 0.387). Even though the severity of pediatric trauma patients increased by the establishment of the trauma center, the mortality rate did not increase further by treating patients with the presence of dedicated attending trauma physician and trauma ICU availability, and daily monitoring for treatment. Published papers to date showed overall mortality ranges in pediatric thoracic trauma from 5 to 30% [1, 21]. In addition, Skinner et al. reported overall mortality of pediatric thoracic trauma in the level 1 trauma center with 16.7%. Therefore, in our study, mortality of 14.9% somewhat acceptable [22].

For now, the regional trauma center managed such pediatric trauma patient with trauma team (trauma surgery and other medical specialties) that can respond as soon as possible upon activation of the trauma team. It is a facility capable of immediately providing integrated and essential treatments, ranging from procedures to surgery [19]. Of course, conservative treatment is more common than surgical treatment, due to the nature of pediatric thoracic trauma, but an appropriate pediatric trauma treatment is still required. In addition, the frequency is very low but critical in the pediatric thoracic trauma area, the need for surgery that requires a high degree of expertise, such as immediate percutaneous vascular procedure or cardiovascular surgery, will increase.

### The need for a pediatric trauma center establishment

Recently, establishment of nationwide system and recognition of the importance of regional trauma centers have been emerged in Korea, however, there is a lack of research on clinical importance and definitive roles of the pediatric trauma centers. In Korea, with a population of more than 50 million, there are 11 hospitals with pediatric intensive care facilities nationwide, of which 97 beds are in the metropolitan area (Seoul city) and only 54 beds in the other all provinces. Some hospitals do not have pediatric intensive care specialists. Similarly, there is no pediatric trauma center at all. However, as a national project, a trauma center was established, and trauma surgeons actively trained and treated not only adults but also pediatric trauma. The trauma center established in this hospital did not ready for pediatric trauma from the beginning. In the past, due to the lack of resources in this tertiary hospital, there were often patients who could not be transported and received appropriate treatment even if a severe pediatric trauma patient occurred. However, as the KMHW supported the trauma center, severe pediatric trauma patients were transferred to this regional trauma center and treatment began. Therefore, if a pediatric trauma center with an organized training system and certification supported by the government is organized within the trauma center, it can show better results in pediatric trauma treatment. Sathya et al. identified in-hospital mortality differences among trauma center type. Crude mortality rates were 2.3% for children treated at adult trauma centers, 1.8% for children treated at mixed trauma centers, and 0.6% for children treated at pediatric trauma centers [23]. Therefore, national efforts should be continued to improve efficiency of establishing the pediatric trauma center in the level 1 regional trauma center, which is to reduce preventable mortality rate, and disability from pediatric trauma through continuous review about treatment results of pediatric patients. These efforts will make our results better.

The main limitation of this study is that it is retrospective. Confounders are the main concern, particularly some concomitant injuries. A well-controlled prospective study may help to answer these questions in the future. The second limitation of this study is that it was not carried out in multiple centers, but was limited to one regional emergency center for 12 years; thus, the data and conclusion derived from the results were somewhat limited. The third limitation is patient who was dead on arrival was not considered for trauma outcome for this study. Autopsy in Korea is extremely limited, so that case was excluded from our data. The final limit is the deaths outside of the emergency center after discharge have not been accounted for in this study.

## Conclusion

In conclusion, although the clinical features of pediatric thoracic trauma are different from those of adults, understanding the anatomical features of the pediatric thorax and the accompanying clinical features will help to increase the survival rate and improve treatment results of children with chest injury in the future. In addition, since the regional trauma center was established, the severity of pediatric thoracic trauma is increasing. Therefore, setting up the pediatric trauma center in the level 1 regional trauma center can improve the outcome of pediatric chest trauma as seen from the results of regional trauma center.

## Supplementary Information

Below is the link to the electronic supplementary material.

Supplementary file1 (DOCX 15 KB)
