# Supplementary material for: Characteristics of pediatric thoracic trauma: in view of before and after the establishment of a regional trauma center
Source: Eur J Trauma Emerg Surg. 2021 Apr 3;48(1):195–204. doi: 10.1007/s00068-021-01658-4 (PMC8019336; doi:10.1007/s00068-021-01658-4)
Supplement: Supplementary file 1 — Supplementary file1 (DOCX 15 KB) [file 68_2021_1658_MOESM1_ESM.docx]

**Appendix 1**

**<Trauma team activation criteria>**

1. Physiologic criteria

A. Airway obstruction/respiratory failure

B. Intubated status before trauma center arrival

C. Adults: respiratory rate<10 breaths/min or>30 breaths/min

D. Adults: systolic blood pressure<90 mmHg

E. Adults: heart rate>100 beats/min

F. Glasgow coma scale<13

2. Anatomical criteria

A. All penetrating injuries

I. Head and neck, chest, abdomen

II. Extremity: proximal to elbow or knee

B. Chest

I. Flail chest

C. Nervous system

I. Open or depressed skull fracture

II. Paralysis or suspected spinal cord injury

D. Extremity and pelvis

I. Pelvic bone fracture

II. Two or more proximal long-bone fractures

III. Crushed, degloved, mangled, or pulseless extremity

IV. Amputation proximal to the wrist or ankle

3. Mechanism of injury

A. Automobile crash: death in same passenger compartment

B. Automobile crash: ejection from automobile

C. Automobile crash>60km/h

D. Automobile versus pedestrian injury>30 km/h

E. Time for evacuation of victim in automobile>20min

(Intrusion, including roof: > 30 cm any site)

F. Motorcycle, bicycle crash > 30km/h

G. Fall

I. Adults: >6m

II. Children: >3m

H. Injury from explosion

4. Attending trauma physician’s judgment

* The trauma team should be activated when the patient has more than one criterion.

* The trauma team activation is determined by the findings observed at the beginning of the patient’s visit.
